# Sex-Dependent Social and Repetitive Behavior and Neurochemical Profile in Mouse Model of Autism Spectrum Disorder

**DOI:** 10.3390/metabo12010071

**Published:** 2022-01-12

**Authors:** Helena Ferreira, Ana Catarina Sousa, José Sereno, João Martins, Miguel Castelo-Branco, Joana Gonçalves

**Affiliations:** 1 Faculty of Medicine, University of Coimbra, 3000-548 Coimbra, Portugal; helenamtferreira.1998@gmail.com; 2 Coimbra Institute for Biomedical Imaging and Translational Research (CIBIT), University of Coimbra, 3000-548 Coimbra, Portugal; josesereno@uc.pt (J.S.); joao.martins@icnas.uc.pt (J.M.); 3 Faculty of Sciences and Technology, University of Coimbra, 3000-370 Coimbra, Portugal; anabsousa98@gmail.com; 4 Institute of Nuclear Sciences Applied to Health (ICNAS), University of Coimbra, 3000-548 Coimbra, Portugal

**Keywords:** autism spectrum disorder, hippocampus, prefrontal cortex, social behavior, repetitive/restrictive behavior

## Abstract

Autism spectrum disorder (ASD) is a neurodevelopmental condition characterized by deficits in social interaction, impaired communication, and repetitive behaviors. ASD presents a 3:1 ratio of diagnosed boys and girls, raising the question regarding sexual dimorphic mechanisms underlying ASD symptoms, and their molecular basis. Here, we performed in vivo proton magnetic resonance spectroscopy in juvenile male and female *Tsc2^+/^^−^* mice (an established genetic animal model of ASD). Moreover, behavior and ultrasonic vocalizations during social and repetitive tasks were analyzed. We found significant sexual dimorphisms in the levels of metabolites in the hippocampus and prefrontal cortex. Further, we observed that female mutant animals had a differential social behavior and presented an increase in repetitive behavior. Importantly, while mutant females displayed a more simplified communication during social tasks, mutant males exhibited a similar less complex vocal repertoire but during repetitive tasks. These results hint toward sex-dependent alterations in molecular and metabolic pathways, which can lead to the sexual dimorphic behaviors and communication observed in social and repetitive environments.

## 1. Introduction

Autism spectrum disorder (ASD) is a neurodevelopmental condition affecting 1 in 59 children [1]. Its diagnosis requires the individual to present deficiencies in social communication and interaction, and restricted/repetitive behaviors (RRB) [2]. Interestingly, ASD presents a male bias, with a male to female ratio of 3:1 [3]. However, the underlying mechanisms for sex bias in ASD remain largely unknown. Therefore, it is important to explore the effect of biological sex on ASD-related pathways and consequent behavioral phenotypes.

Deficiencies in social interaction are widely reported in ASD patients (e.g., [4,5,6,7,8]), as well as in animal models of the disorder, who present reduced interaction, abnormal social preference, and lack of preference for social novelty (e.g., [9,10,11,12,13,14]). Moreover, patients with ASD present stereotyped, ritualized movements and patterns of behavior, and fixated interests, with an abnormal intensity and concentration [2]. These RRB can be assessed in rodents via analysis of persistent, stereotyped activities such as self-grooming or marble burying [15]. Several authors have reported increased levels of self-grooming behavior [12,16,17,18,19,20,21,22,23,24,25,26] and of marble-burying activity [22,23,27,28,29] in syndromic and environmental mouse models of ASD.

Further, the abnormalities of speech produced by ASD individuals [30] reveal impaired communication as a hallmark of the disease. Rodents are able to communicate through ultrasonic vocalizations (USVs), which are produced by juvenile and adult animals in social contexts [15]. Importantly, USVs have also been observed in non-social situations, such as during the exploration of a novel environment or as a consequence of stress [31,32]. Several works have revealed abnormal communication in animal models of ASD (e.g., [11,33,34,35,36,37,38]), even though the molecular mechanisms underlying this manifestation also remain unknown.

Alterations in prefrontal cortex (PFC) circuits are reported to cause social deficiencies [39,40,41], while deficient hippocampal structure and function are related to abnormal cognitive mechanisms [42]. Indeed, an aluminum-induced model of cholinergic deficiency presented altered acetylcholine levels in the PFC and hippocampus, along with reduced social skills and cognitive function [43]. Further, reduced levels of myo-inositol in the PFC were reported in rats exhibiting high-impulsivity behavior [44], as well as increased lactate in socially isolated rats [45]. These results underscore the role of specific brain structures and metabolites in the development of ASD manifestations. However, scarce studies address both the behavior and metabolic hallmarks of ASD—namely, altered social and stereotyped behaviors and specific molecular and metabolic signatures in ASD individuals, and only a few dispersed connections have been described, especially regarding biological sex. 

Here, we performed in vivo proton magnetic resonance spectroscopy to analyze the levels of several metabolites in the PFC and hippocampus, in juvenile male and female mice, using a genetic model of ASD, tuberous sclerosis complex 2 (*Tsc2^+/^^−^*) mice. Additionally, we carried social and RRB-eliciting tasks. We found altered cortical gamma-aminobutyric acid (GABA)/glutamate (Glu) ratio in mutant females. Further, it was observed sexual dimorphisms in the levels of total choline (tCho), N-acetyl-aspartate + N-acetyl-aspartyl-glutamate (NAA + NAAG), taurine (Tau), alanine (Ala), and inositol (Ins) in the hippocampus and prefrontal cortex (PFC). These neurometabolic alterations may be somehow associated with the sex-dependent behaviors and communication found in both social and RRB-eliciting tasks, thus deserving further research. 

## 2. Results

### 2.1. Spectroscopic Analyses

To identify specific neurochemical profiles in male and female *Tsc2^+/^^−^* mice, we performed in vivo proton magnetic resonance spectroscopy in both hippocampus and PFC. The metabolites analyzed are listed in Supplementary Tables S1 and S2. 

**Figure 1:**
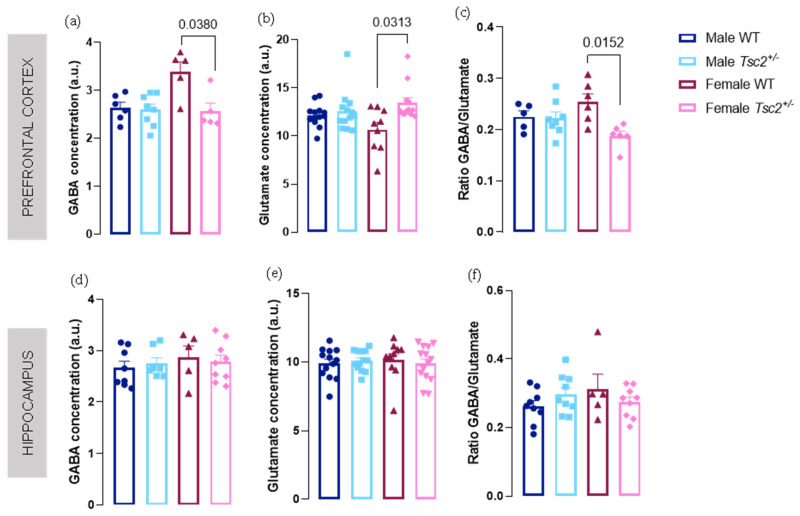
Excitation/inhibition balance is disturbed in a sex- and region-dependent manner. ^1^H-magnetic resonance spectroscopy (MRS) analysis of concentration levels of cortical levels of (**a**) GABA and (**b**) glutamate were decreased and increased, respectively, in female *Tsc2^+/−^*. Accordingly, (**c**) GABA/glutamate ratio in the prefrontal cortex was altered. (**d**–**f**) In the hippocampus, MRS evaluation showed no changes between sex and genotypes. The results are expressed as mean ± SEM. Prefrontal cortex GABA: 24 animals (6 male WT, 8 male *Tsc2^+/^^−^*, 5 female WT, 5 female *Tsc2^+/^^−^*); prefrontal cortex glutamate: 47 animals (13 male WT, 13 male *Tsc2^+/^^−^*, 9 female WT, 12 female *Tsc2^+/^^−^*); prefrontal cortex GABA/glutamate ratio: 25 animals (5 male WT, 8 male *Tsc2^+/^^−^*, 6 female WT, 6 female *Tsc2^+/^^−^*); hippocampus GABA: 29 animals (8 male WT, 7 male *Tsc2^+/^^−^*, 5 female WT, 9 female *Tsc2^+/^^−^*); hippocampus glutamate: 50 animals (13 male WT, 13 male *Tsc2^+/^^−^*, 10 female WT, 14 female *Tsc2^+/^^−^*); hippocampus GABA/glutamate ratio: 32 animals (9 male WT, 9 male *Tsc2^+/^^−^*, 5 female WT, 9 female *Tsc2^+/^^−^*). Statistical analysis was performed using Kruskal–Wallis, followed by Dunn’s multiple comparison test.

In the PFC, neurotransmitters Glu and GABA were altered but only in females (Figure 1). In fact, we found an up- and downregulation of levels of Glu and GABA, respectively, in female *Tsc2^+/^^−^* (Glu: female WT = 10.66 ± 0.7597 vs. female *Tsc2^+/^^−^* = 13.43 ± 0.5382; *p* = 0.0313; GABA: female WT = 3.383 ± 0.2118 vs. female *Tsc2^+/^^−^* = 2.565 ± 0.1685; *p* = 0.0380; Figure 1a,b). Accordingly, GABA/Glu ratio was significantly altered with a decrease in transgenic females (female WT = 0.2537 ± 0.01624 vs. female *Tsc2^+/^^−^* = 0.1878 ± 0.009485; *p* = 0.0152; Figure 1c). No significant differences were found concerning these metabolites in the hippocampus (Figure 1d–f).

**Figure 2:**
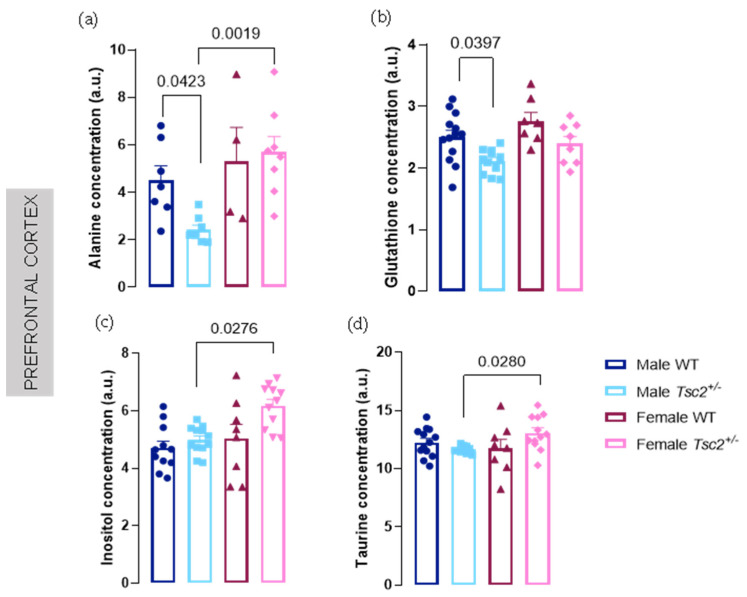
*Tsc2^+/^^−^* leads to sex-dependent cortical metabolite alterations. ^1^H-magnetic resonance spectroscopy (MRS) analysis demonstrated a down-regulation of concentration levels of (**a**) alanine and (**b**) glutathione in male *Tsc2^+/^^−^*, compared with their littermates wild-type (WT). Additionally, it was observed a reduction in (**c**) inositol and (**d**) taurine in male transgenic mice but in comparison with transgenic females. The results are expressed as mean ± SEM. Alanine: 27 animals (7 male WT, 8 male *Tsc2^+/^^−^*, 4 female WT, 8 female *Tsc2^+/^^−^*); glutathione: 39 animals (13 male WT, 11 male *Tsc2^+/^^−^*, 7 female WT, 8 female *Tsc2^+/^^−^*); inositol: 42 animals (11 male WT, 12 male *Tsc2^+/^^−^*, 8 female WT, 11 female *Tsc2^+/^^−^*); taurine: 43 animals (13 male WT, 10 male *Tsc2^+/^^−^*, 8 female WT, 12 female *Tsc2^+/^^−^*). Statistical analysis was carried out using Kruskal–Wallis, followed by Dunn’s multiple comparison test.

Regarding other metabolites, we observed a decrease in Ala and glutathione (GSH) levels in male *Tsc2^+/^^−^* (Ala: male WT = 4.517 ± 0.6063 vs. male *Tsc2^+/^^−^* = 2.427 ± 0.1913; *p* = 0.0423, Figure 2a; GSH: male WT = 2.501 ± 0.1113 vs. male *Tsc2^+/^^−^* = 2.106 ± 0.06063; *p* = 0.0397; Figure 2b). For Ala levels, sexual dimorphism is confirmed when comparing transgenic males and females (male *Tsc2^+/^^−^* = 2.427 ± 0.1913 vs. female *Tsc2^+/^^−^* = 5.685 ± 0.6611; *p* = 0.0019; Figure 2a). Furthermore, sex differences were also detected for cortical Ins and Tau where transgenic females exhibited increased levels, compared with transgenic males (Ins: male *Tsc2^+/^^−^* = 4.988 ± 0.1379 vs. female *Tsc2^+/^^−^* = 6.174 ± 0.2288; *p* = 0.0276, Figure 2c; Tau: male *Tsc2^+/^^−^* = 11.65 ± 0.09962 vs. female *Tsc2^+/^^−^* = 13.07 ± 0.4239; *p* = 0.0280, Figure 2d).

**Figure 3:**
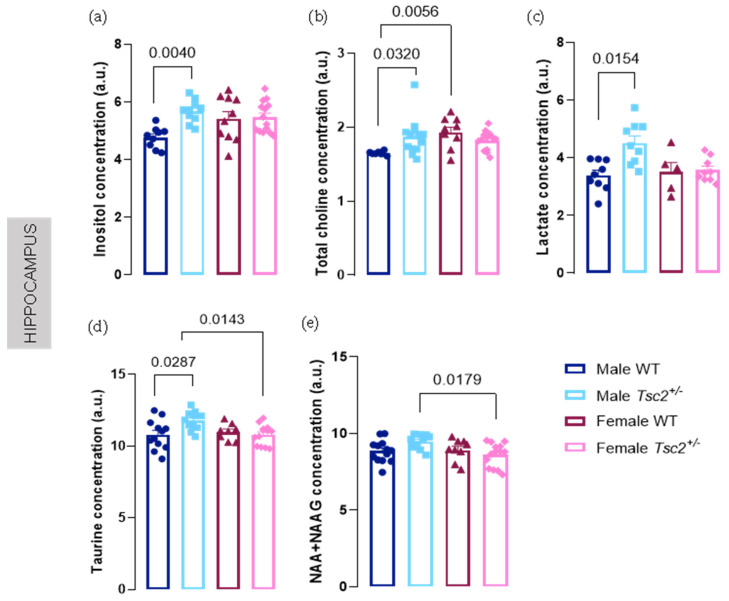
Hippocampal neurochemical profile of male *Tsc2^+/^^−^* mice was altered. It was found that concentration levels of (**a**) total choline, (**b**) inositol, (**c**) lactate, (**d**) taurine, and (**e**) NAA + NAAG were augmented using ^1^H-magnetic resonance spectroscopy (MRS). The results are expressed as mean ± SEM. Inositol: 43 animals (9 male WT, 10 male *Tsc2^+/^^−^*, 10 female WT, 14 female *Tsc2^+/^^−^*); total choline: 42 animals (7 male WT, 13 male *Tsc2^+/^^−^*, 9 female WT, 13 female *Tsc2^+/^^−^*); lactate: 32 animals (9 male WT, 9 male *Tsc2^+/^^−^*, 5 female WT, 9 female *Tsc2^+/^^−^*); taurine: 45 animals (12 male WT, 12 male *Tsc2^+/^^−^*, 8 female WT, 13 female *Tsc2^+/^^−^*); NAA + NAAG: 46 animals (13 male WT, 10 male *Tsc2^+/^^−^*, 9 female WT, 14 female *Tsc2^+/^^−^*). Statistical analysis was carried out using Kruskal–Wallis, followed by Dunn’s multiple comparison test.

Regarding hippocampal neurochemical profile, most significant differences were observed in males. In fact, *Tsc2^+/^^−^* males showed increased levels of various metabolites such as tCho (male WT = 1.651 ± 0.00989 vs. male *Tsc2^+/^^−^* = 1.874 ± 0.0698; *p* = 0.0323, Figure 3a), Ins (male WT = 4.768 ± 0.1217 vs. male *Tsc2^+/^^−^* = 5.674 ± 0.1246; *p* = 0.0040, Figure 3b), lactate (Lac) (male WT = 3.393 ± 0.177 vs. male *Tsc2^+/^^−^* = 4.513 ± 0.2463; *p* = 0.0154, Figure 3c), and Tau (male WT = 10.81 ± 0.2973 vs. male *Tsc2^+/^^−^* = 11.79 ± 0.1872; *p* = 0.0287, Figure 3d). Regarding females, no changes were found in any of these metabolites in the hippocampus when we compared transgenic with WT littermates. Confirming sexual dimorphism among transgenic mice, we observed reduced NAA + NAAG and Tau levels in females, compared with males (NAA + NAAG: male *Tsc2^+/^^−^* = 9.479 ± 0.1503 vs. female *Tsc2^+/^^−^* = 8.603 ± 0.2047; *p* = 0.0179, Figure 3e; Tau: male *Tsc2^+/^^−^* = 11.79 ± 0.1872 vs. female *Tsc2^+/^^−^* = 10.80 ± 0.1976; *p* = 0.0143, Figure 3d).

### 2.2. Behavioral Testing

To test whether the observed metabolic profile was accompanied by core ASD manifestations in *Tsc2^+/^^−^* mice, we performed behavioral tests, namely, social behavioral test and RRB-eliciting test.

#### 2.2.1. Social Behavioral Test

**Figure 4:**
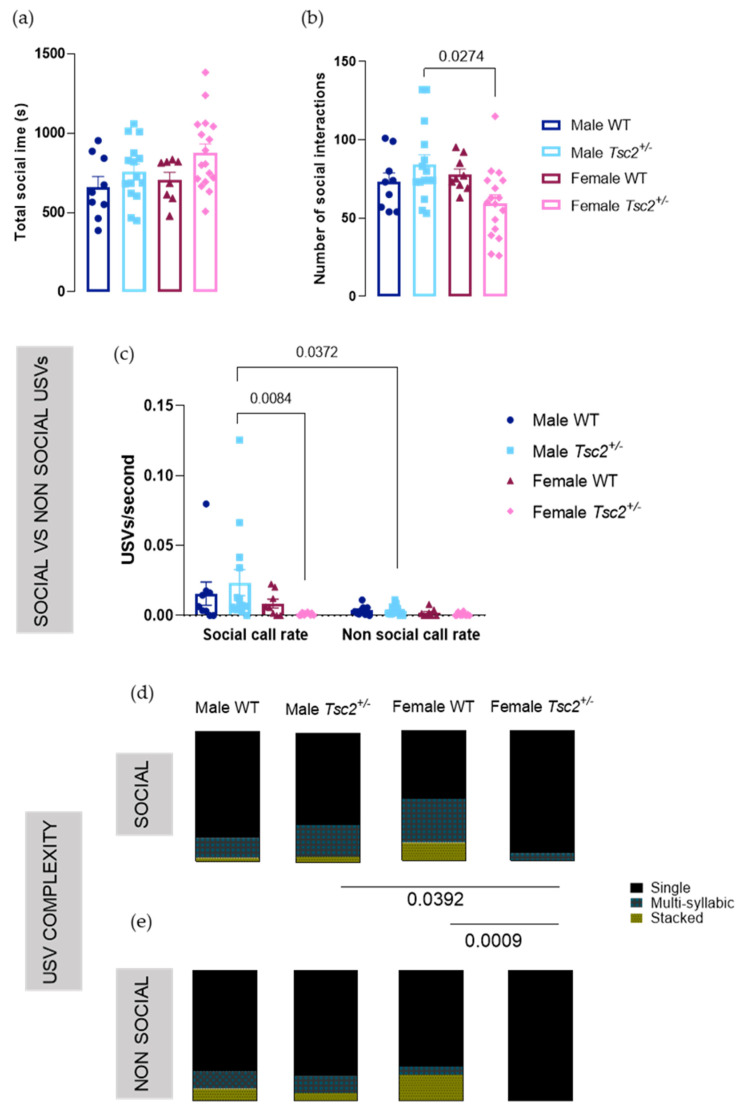
Female *Tsc2^+/^^−^* mice showed social-communication skill impairment. Although changes were not found in (**a**) total social time between animal groups, transgenic females display a reduction in (**b**) number of social interactions. (**c**) Social and non-social call rates (USVs/second) showed decreased vocalization rate in transgenic females. Discrimination of USVs categories into (**d**) single, multi-syllabic, and stacked USVs produced during social behavior, and (**e**) single, multi-syllabic, and stacked USVs produced during non-social behavior, revealed decreased vocal complexity in mutant females. The results are expressed as mean ± SEM from 50 pairs of animals (9 male wild-type (WT) pairs, 15 male *Tsc2^+/^^−^* pairs, 9 female WT pairs, 17 female *Tsc2^+/^^−^* pairs). Statistical analysis was performed using Kruskal–Wallis, followed by Dunn’s multiple comparison test and two-way ANOVA, followed by Sidak’s multiple comparison test.

Since impaired social behavior is a hallmark of ASD, we performed a juvenile social play test. Transgenic females tend to socialize longer, although no statistically significance was found (females WT = 704.8 ± 47.86s vs. females *Tsc2^+/^^−^* = 877.0 ± 56.08s; *p* = 0.4576; Figure 4a). Interestingly, we found a decreased number of social interactions in *Tsc2^+/^^−^* females, compared with males (males *Tsc2^+/^^−^* = 84.07 ± 6.330 interactions vs. females *Tsc2^+/^^−^* = 59.53 ± 5.398 interactions; *p* = 0.0274; Figure 4b), revealing that transgenic females prefer less and longer interactions, which may be considered an example of sexual dimorphic social behavior. 

As both behavior and USVs were recorded simultaneously, we were able to investigate vocal performance during social vs. non-social behavior. We found no significant differences regarding USV length, principal frequency, or frequency amplitude (Supplementary Figure S1a–c). However, analysis of the call rate during social and in non-social behaviors showed a significant genotype effect (F (3, 84) = 3214; *p* = 0.0270) and behavior effect (F (1, 84) = 7982; *p* = 0.0059). Mutant males vocalized with an increased call rate in social environments than during non-social behavior (social call rate = 0.04357 ± 0.02180 USVs/s vs. non-social call rate = 0.003634 ± 0.0008391 USVs/s; *p* = 0.0372). Importantly, we observed that mutant females vocalized with a significantly decreased call rate when socializing (male *Tsc2^+/^^−^* = 0.04357 ± 0.02180 USVs/s vs. female *Tsc2^+/^^−^* = 0.00106 ± 0.000259 USVs/s; *p* = 0.0084).

When we discriminated the type of USV emitted during social behavior, there was a significant interaction between call category and genotype (F (6, 96) = 4615; *p* = 0.0004) and an effect of call category (F (2, 96) = 109,5; *p* < 0.0001). We found that female *Tsc2^+/^^−^* presented deficiencies in their vocal repertoire, as they produced significantly more of the less complex USV class, single USVs, not producing stacked USVs at all, unlike all other experimental groups (*Tsc2^+/^^−^*: single USVs male *Tsc2^+/^^−^* = 70.67 ± 7.244% vs. single USVs female *Tsc2^+/^^−^* = 94.44 ± 5.556%; *p* = 0.0392; females: single USVs female WT = 52.66 ± 14.05%; single USVs female *Tsc2^+/^^−^* = 94.44 ± 5.556%; *p* = 0.0009; Figure 4d). Indeed, such vocal deficiencies are also present during non-social behaviors, during which transgenic females only produced single USVs; however, no significant differences were found (Figure 4e). 

Overall, these results indicate a differential social interaction behavior in female *Tsc2^+/^^−^*, who also present vocalization deficiencies during such behavior. It is also interesting to note the sexual dimorphism observed in vocalization complexity, where no differences were found between males.

#### 2.2.2. RRB Behavioral Test

**Figure 5:**
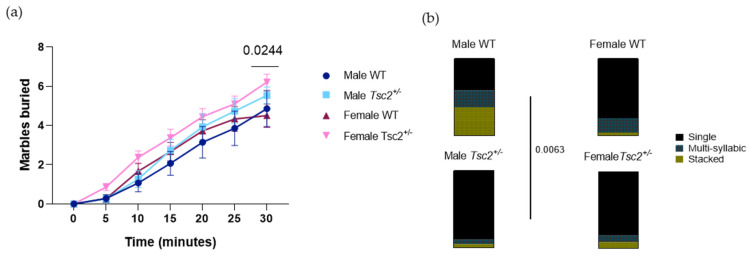
Female *Tsc2^+/^^−^* mice showed increased repetitive behavior: (**a**) total number of buried marbles shows an increase in transgenic females; (**b**) analysis on communication complexity during RRB-eliciting task with discrimination of USVs categories into single, multi-syllabic, and stacked USVs shows impairment in mutant males. The results are expressed as mean ± SEM from 77 animals (14 male WT, 15 male *Tsc2^+/^^−^*, 18 female WT, 30 female *Tsc2^+/^^−^*). Statistical analysis was performed using two-way ANOVA, followed by Sidak’s multiple comparison test.

RRBs are among the core symptoms of ASD and are widely observed in ASD animal models. Here, we observed an increased number of total buried marbles by female *Tsc2^+/^^−^* (female WT = 4.500 ± 0.5955 marbles buried; female *Tsc2^+/^^−^* = 6.207 ± 0.4043 marbles buried; *p* = 0.0244; Figure 5a). These results indicate an increased repetitive, stereotyped behavior displayed by transgenic females. Regarding USV production, there were no significant differences regarding USV length, principal frequency, or frequency amplitude (Supplementary Figure S1d–f). However, the analysis on the complexity of the USVs produced during this task revealed that differences in the vocal repertoire were found between WT and transgenic males but not females. There was a significant interaction between call category and genotype (F (6, 57) = 3666; *p* = 0.0038) and an effect of call category (F (2, 57) = 46,13; *p* < 0.0001). Indeed, *Tsc2^+/^^−^* males emitted significantly more single USVs than their WT littermates (male WT = 41.19 ± 14.65%; male *Tsc2^+/^^−^* = 89.52 ± 6.801%; *p* = 0.0063), thus displaying reduced vocal complexity (Figure 5b). This seems to indicate that, contrary to social tasks, RRB-eliciting tasks promote alterations in vocal complexity in *Tsc2^+/^^−^* males.

## 3. Discussion

Studies on region-specific metabolic profiles in ASD poorly address the behavioral phenotypes of the condition. Importantly, most studies carried out thus far give little-to-no relevance to biological sex. Here, we investigated in detail the metabolic signatures of the hippocampus and PFC of male and female *Tsc2^+/^^−^* mice, an established genetic model of ASD. We found region-specific sexual dimorphisms in several metabolites. Indeed, an increase in Ala, Tau, and Ins levels in the PFC and a decrease in NAA + NAAG and Tau levels in the hippocampus of mutant females were observed. Additionally, while transgenic males differed in cortical Ala and GSH and hippocampal tCho, Ins, Lac, and Tau, transgenic females showed important differences in cortical GABA/Glu ratio relative to WT. 

Given these results, we performed behavioral and communication analyses in another set of juvenile mice to investigate whether the alterations observed in the brain translated into abnormal phenotypes. In this sense, we carried out social and repetitive tasks, to investigate into core features of ASD. Moreover, we recorded the ultrasonic vocalizations produced by the animals during the above-mentioned tasks to assess communication ability. The classification algorithm used [46] allows the determination of vocal complexity: Single USVs are the least intricate vocalizations, not having sudden frequency changes, such as multi-syllabic USVs, or simultaneous independent sounds at different frequencies such as stacked USVs. Our data indicate that transgenic females showed altered sociability, as well as decreased vocal complexity, hinting that altered social behavior may promote or be linked to vocal alterations as well. 

In this work, the phenotypic disturbances displayed by transgenic females were also present in RRB-eliciting tasks. RRBs are associated with anxiety [47,48,49], as sameness of behavior may represent a strategy to better control the individual’s surroundings and avoid uncertainty, and thus temporarily reduce anxious feelings [50]. Interestingly, the marble-burying test, such as the one performed in this work, has shown controversy, as some reports have described reduced and not increased burying activity [24,51,52,53]. However, this behavior may relate to the avoidance of novel objects [54]. Therefore, we posit that the altered sociability of transgenic females may also represent a stress-coping strategy. In fact, increased anxiety paired with reduced hippocampal NAA + NAAG in *Tsc2^+/^^−^* females is in line with previous findings, in which NAA levels diminished in the hippocampus following fear conditioning [55]. A reduction in the levels of this metabolite in the hippocampus and cortex after an acute cold swim stress test has also been described [56].

Importantly, even though mutant females also displayed increased repetitive, stereotyped, anxious behavior, this was not accompanied by alterations in communication in RRB-eliciting tasks. Indeed, while social tasks elicited communication differences between females, RRB-eliciting tasks promoted communication discrepancies between males. It has been described that the USV call rate of male adult mice is influenced by social isolation and is dependent on behavioral states [57]. Interestingly, a study using only female CBA/CaJ mice showed that social isolation alters USV perception, also indicating that vocal communication may be dependent on experience and/or emotional state [58]. However, currently, a gap exists regarding USV production during RRB-eliciting tasks, as most studies focus on neonatal age or social contexts. Our data give important insights into the factors that may be governing communication and suggest that they could be sex dependent as well as environment specific. 

Interestingly, our results demonstrate an overall greater severity in ASD-like phenotype in mutant females, rather than males. This seems to contradict the notion that males are more affected by the disorder, both by prevalence and by liability, as there seem to exist sex-differential factors that may potentiate risk in males and protect females [59]. However, this does not appear to be the case when ASD in TSC individuals is specifically considered [60]. Indeed, the syndrome was observed to be more pervasive in females, both in humans [61] and in animal studies [62]. This is in accordance with our results and further validates our animal model for the research of ASD in TSC individuals. 

Region-specific expression patterns of metabolic proteins have already been described in the PFC and hippocampus of male mice susceptible and not susceptible to social stress. This indicates that specific cortical and hippocampal metabolic signatures may be underlying sociability and anxiety [63]. Indeed, region-specific mechanisms of excitation/inhibition imbalance have been previously described [64]. This is in line with our results, which extend this matter by considering biological sex. Indeed, we found genotype-dependent differential GABA/Glu ratio in the PFC only in females, who also display differences in repetitive behavior and in social behavior and communication. This suggests that a sex-dependent and increased cortical excitatory tone may be driving the observed core features of ASD and their corresponding sexual dimorphisms in this particular period of neurodevelopment. 

Region-specific alterations seem to be associated with distinct behavioral outcomes. While hippocampal studies demonstrate that metabolic alterations or deficits result in depressive symptoms and learning deficiencies, studies on cortical metabolic profiles show that altered metabolite changes result in altered social, impulsive, or anxious behavior. In the hippocampus, chronic restraint stress has been shown to alter cholinergic signaling [65], and conversely, choline supplementation was beneficial for the reversal of nicotine-induced learning deficits [66]. Further, treatments with creatine and taurine yielded antidepressant effects, with increased hippocampal catecholamine and serotonin levels [67]. This is in agreement with our data on taurine levels, where transgenic females displayed a reduction in the hippocampus and decreased vocal complexity, which could be linked to cognitive disabilities. Interestingly, transgenic females showed increased taurine levels in the prefrontal cortex, which indicates that behavioral phenotypes are dependent on the metabolic profiles of a specific brain region. Additionally, a study found that caffeine plus taurine supplementation resulted in greater activity [68]; in this sense, the increased hippocampal taurine in transgenic males we observed could be translated into their higher number of social interactions. On the other hand, this augmented activity could be an expression of increased hyperactivity caused by decreased inositol in the prefrontal cortex of transgenic males, as previously shown [44]. Moreover, a study on mice with impaired glutathione synthesis found an association between decreased myo-inositol concentration and social isolation [69]. This is in accordance with our findings, as transgenic females showed increased inositol levels and sociability.

Overall, this work sheds light on the sexually dimorphic, region-specific metabolic profiles that may be mediating sex-dependent behaviors and communication displayed in different environments. More studies focusing on the role of biological sex are necessary in order to further understand how male and female metabolic signatures differ, and how that translates into abnormal ASD-like behavior. 

## 4. Materials and Methods

### 4.1. Animals

*Tsc2^+/^^−^* mice were generated and backcrossed to a C57BL/6N. Mice for experiments were generated by crossing *Tsc2^+/^^−^* animals with mice with C57BL/6J background. All pups were housed together with the dam until postnatal day 21 (P21) and segregated by sex from P21 onwards. All animals were maintained in a housing room with a 12 h light–12 h dark cycle, at 21 ± 2 °C. Identification of pups was performed at P4 with permanent tattoos on the toes. Tail tips were collected at P4 for posterior genotyping. All experiments were carried out in accordance with the European Union Council Directive (2010/63/EU) and the National Regulations, approved by the Internal Review Board of ICNAS and conducted under the authority of the Project License (1/2017).

### 4.2. Magnetic Resonance Spectroscopy

**Figure 6:**
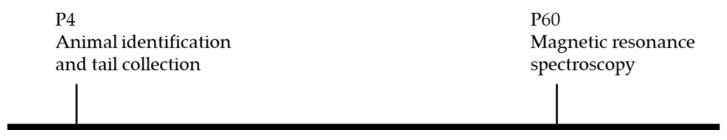
Timeline of resonance spectroscopy experiments in this study. Animals were identified on postnatal day (P)4 with permanent tattoos on toes and tails were collected for genotyping. All animals and went through localized ^1^H-MRS on P60.

For localized ^1^H-magnetic resonance spectroscopy (^1^H-MRS), data were collected in a volume of interest placed on the hippocampus and pre-frontal cortex (PFC). B0 map was acquired before spectroscopy, and shims were optimized through a MAPSHIM voxel. Spectra were acquired using a point-resolved spectroscopy (PRESS) sequence with outer volume suppression (OVS) and VAPOR water suppression [70,71]. The following parameters were used: TR = 2500 ms, TE = 16.225 ms, number of averages = 720, 3 flip angles = 90°, 142°, 142°, bandwidth = 5000 Hz, number of acquired points = 2048 yielding a spectral resolution of 1.22 Hz/pt. The total acquisition time was 30 min. Before each spectrum, we acquired an unsuppressed water spectrum at the same voxel location (TE = 16.225 ms, TR = 2500 ms, 16 averages, scanning time = 40 s) (Supplementary Figure S2). Data analysis of ^1^H-MRS spectra was performed using linear combination modeling LCModel (Stephen Provencher Inc., Toronto, ON, Canada) [72]. Metabolite quantification was performed applying the internal water reference method. Concentrations in millimole units were calculated for metabolites, and results are presented in arbitrary units (a.u.). Only metabolites with Cramér–Rao bounds < 20% were considered for statistical analysis. A timeline of spectroscopic experiments is represented in Figure 6.

### 4.3. Ultrasonic Vocalization Recordings and Analysis

A 55 cm × 50 cm × 70 cm (H × D × W) anechoic chamber was assembled with 1.5 cm thick acrylic sheets and fully covered with absorbing foam on the inside, to block external sound. All USVs were acquired using an ultrasound recording system with Avisoft CM16/CMPA condenser microphone placed 10 cm above container wall top, UltrasoundGate 416H amplifier, and Avisoft Recorder software (Avisoft Bioacoustics, Glienicke/Nordbahn, Germany). Sonograms were generated, with 512 point FFT, 16-bit format, a sampling frequency of 250 kHz, a time resolution of 1 ms, a frequency resolution of 488 Hz, and an overlap of 50%. USVs were further analyzed using MATLAB toolbox DeepSqueak version 2.6.1., which allowed the extraction of individual mouse USV calls by applying the Mouse Call_Network_V2 neural network, with a chunk length analysis of 6 s, an overlap of 0.1 s, a high-frequency cut-off of 125 kHz, and no score threshold. The principal frequency, frequency range, and duration of each call were extracted, and call rate (number of USVs produced over time) was calculated. Each USV was manually classified into three classes. The characteristics of each USV class are as follows: single: one waveform only present in the sonogram; multi-syllabic: two or more waveforms, with no interval in time between them and no temporal overlap; stacked: two or more waveforms with temporal overlap.

### 4.4. Video Recordings

The juvenile play test and marble-burying test were video recorded with a Logitech C170 video camera (Logitech Europe S.A., Lausanne, Switzerland) on top of the chamber under dim red light (5 lux). All video recordings were manually analyzed by the operator. 

### 4.5. Juvenile Social Play Test

**Figure 7:**
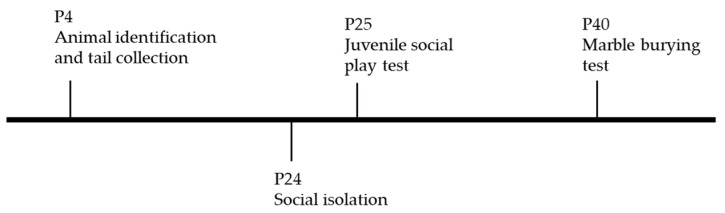
Timeline of behavioral experiments in this study. All animals were identified on postnatal day (P)4 with permanent tattoos on toes, and tails were collected for genotyping. On P24, animals were socially isolated in individual clean cages and juvenile social play test was carried out on P25. On P40, marble-burying test was performed.

On P24, animals were isolated for 24 h prior to the social task and housed in a new clean mouse cage with fresh bedding, to potentiate their social behavior. On P25, two same-sex, same-genotype animals were placed on a clean, standard cage in the anechoic chamber [73]. The vertical walls of the cage were further extended with acrylic sheets to avoid the animal exiting the apparatus. The animals were allowed to interact for 30 min. At the end of the experiment, both animals were returned to their home cage. Video recordings were analyzed by an operator blind to sex and genotype, to identify social behavior. Actions considered as social behaviors included investigative interactions (sniffing, following, mutual circle), affiliative interactions (group sitting, allogrooming, push under), play solicitation (crawl, push past, approach), and agonistic interactions (threat, aggression, defense, flight, submission) [74,75]. Total and average social time were manually tracked with a stopwatch, and the number of social interactions was registered. A timeline of behavioral experiments is represented in Figure 7. 

### 4.6. Marble-Burying Test

On P40, each animal was placed on a clean, standard mouse cage filled with a 5 cm layer of sawdust and with 12 marbles equidistantly placed on top of the sawdust, on a 3 × 4 arrange. The vertical walls of the cage were further extended with the application of acrylic sheets, to avoid the animal exiting the apparatus. The cage was inside the anechoic chamber. The animal was allowed to interact with marbles for 30 min. At the end of the experiment, the animal was returned to its home cage. Video recordings were analyzed by an operator blind to sex and genotype, to register the number of marbles buried at 5, 10, 15, 20, 25, and 30 min of the experiment [76]. A timeline of behavioral experiments is represented in Figure 7. 

### 4.7. Statistical Analyses

All data were analyzed using GraphPad Prism version 8.0.1 (GraphPad Software, San Diego, CA, USA). Outliers were identified as values outside of mean ± 3SD interval and excluded from statistical tests. Two-way ANOVAs followed by Sidak’s multiple comparisons tests (USV analyses) or Kruskal–Wallis, followed by Dunn’s multiple comparison tests (MRS and behavioral analyses), were conducted to compare experimental groups. All effects are reported as significant at *p* < 0.05. Error bars are given as SEM.

## Data Availability

The data presented in this study are available in article or Supplementary Material.

## Supplementary Materials

The following are available online at www.mdpi.com/article/10.3390/metabo12010071/s1, Figure S1: Localized ^1^H NMR voxel in the prefrontal cortex and hippocampus, Figure S2: Length, principal frequency and amplitude–frequency of USVs produced during social behavior task and RRB-eliciting task, Table S1: ^1^H-magnetic resonance spectroscopy (MRS) analysis of cortical neurochemical profile, Table S2: ^1^H-magnetic resonance spectroscopy (MRS) analysis of hippocampal neurochemical profile.

## References

[1] Baio J., Wiggins L., Christensen D.L., Maenner M.J., Daniels J., Warren Z., Kurzius-Spencer M., Zahorodny W., Rosenberg C.R., White T. (2018). Prevalence of Autism Spectrum Disorder Among Children Aged 8 Years—Autism and Developmental Disabilities Monitoring Network, 11 Sites, United States, 2014. MMWR Surveill. Summ..

[2] American Psychiatric Association (2013). DSM-5 Task Force. Diagnostic and Statistical Manual of Mental Disorders.

[3] Loomes R., Hull L., Mandy W.P.L. (2017). What Is the Male-to-Female Ratio in Autism Spectrum Disorder? A Systematic Review and Meta-Analysis. J. Am. Acad. Child Adolesc. Psychiatry.

[4] Whitehouse A.J.O., Durkin K., Jaquet E., Ziatas K. (2009). Friendship, Loneliness and Depression in Adolescents with Asperger’s Syndrome. J. Adolesc..

[5] Durkin K., Conti-Ramsden G. (2010). Young People with Specific Language Impairment: A Review of Social and Emotional Functioning in Adolescence. Child. Lang. Teach. Ther..

[6] Mazurek M.O. (2014). Loneliness, Friendship, and Well-Being in Adults with Autism Spectrum Disorders. Autism.

[7] Lowe J.K., Werling D.M., Constantino J.N., Cantor R.M., Geschwind D.H. (2015). Social Responsiveness, an Autism Endophenotype: Genomewide Significant Linkage to Two Regions on Chromosome 8. Am. J. Psychiatry.

[8] Sedgewick F., Hill V., Yates R., Pickering L., Pellicano E. (2016). Gender Differences in the Social Motivation and Friendship Experiences of Autistic and Non-Autistic Adolescents. J. Autism Dev. Disord..

[9] Kwon C.H., Luikart B.W., Powell C.M., Zhou J., Matheny S.A., Zhang W., Li Y., Baker S.J., Parada L.F. (2006). Pten Regulates Neuronal Arborization and Social Interaction in Mice. Neuron.

[10] Tabuchi K., Blundell J., Etherton M.R., Hammer R.E., Liu X., Powell C.M., Südhof T.C. (2007). A Neuroligin-3 Mutation Implicated in Autism Increases Inhibitory Synaptic Transmission in Mice. Science.

[11] Jamain S., Radyushkin K., Hammerschmidt K., Granon S., Boretius S., Varoqueaux F., Ramanantsoa N., Gallego J., Ronnenberg A., Winter D. (2008). Reduced Social Interaction and Ultrasonic Communication in a Mouse Model of Monogenic Heritable Autism. Proc. Natl. Acad. Sci. USA.

[12] Peça J., Feliciano C., Ting J.T., Wang W., Wells M.F., Venkatraman T.N., Lascola C.D., Fu Z., Feng G. (2011). Shank3 Mutant Mice Display Autistic-like Behaviours and Striatal Dysfunction. Nature.

[13] Gkogkas C.G., Khoutorsky A., Ran I., Rampakakis E., Nevarko T., Weatherill D.B., Vasuta C., Yee S., Truitt M., Dallaire P. (2013). Autism-Related Deficits via Dysregulated EIF4E-Dependent Translational Control. Nature.

[14] Clipperton-Allen A.E., Page D.T. (2014). Pten Haploinsufficient Mice Show Broad Brain Overgrowth but Selective Impairments in Autism-Relevant Behavioral Tests. Hum. Mol. Genet..

[15] Pasciuto E., Borrie S.C., Kanellopoulos A.K., Santos A.R., Cappuyns E., D’Andrea L., Pacini L., Bagni C. (2015). Autism Spectrum Disorders: Translating Human Deficits into Mouse Behavior. Neurobiol. Learn. Mem..

[16] McFarlane H.G., Kusek G.K., Yang M., Phoenix J.L., Bolivar V.J., Crawley J.N. (2008). Autism-like Behavioral Phenotypes in BTBR T+tf/J Mice. Genes Brain Behav..

[17] Gilbert J., O’Connor M., Templet S., Moghaddam M., Di Via Ioschpe A., Sinclair A., Zhu L.Q., Xu W., Man H.Y. (2020). NEXMIF/KIDLIA Knock-out Mouse Demonstrates Autism-Like Behaviors, Memory Deficits, and Impairments in Synapse Formation and Function. J. Neurosci..

[18] Jones K.L., Pride M.C., Edmiston E., Yang M., Silverman J.L., Crawley J.N., Van de Water J. (2020). Autism-Specific Maternal Autoantibodies Produce Behavioral Abnormalities in an Endogenous Antigen-Driven Mouse Model of Autism. Mol. Psychiatry.

[19] Etherton M.R., Blaiss C.A., Powell C.M., Südhof T.C. (2009). Mouse Neurexin-1α Deletion Causes Correlated Electrophysiological and Behavioral Changes Consistent with Cognitive Impairments. Proc. Natl. Acad. Sci. USA.

[20] Pobbe R.L.H., Pearson B.L., Defensor E.B., Bolivar V.J., Blanchard D.C., Blanchard R.J. (2010). Expression of Social Behaviors of C57BL/6J versus BTBR Inbred Mouse Strains in the Visible Burrow System. Behav. Brain Res..

[21] Carter M.D., Shah C.R., Muller C.L., Crawley J.N., Carneiro A.M.D., Veenstra-VanderWeele J. (2011). Absence of Preference for Social Novelty and Increased Grooming in Integrin Β3 Knockout Mice: Initial Studies and Future Directions. Autism Res..

[22] Amodeo D.A., Jones J.H., Sweeney J.A., Ragozzino M.E. (2012). Differences in BTBR T+ Tf/J and C57BL/6J Mice on Probabilistic Reversal Learning and Stereotyped Behaviors. Behav. Brain Res..

[23] Camacho J., Jones K., Miller E., Ariza J., Noctor S., Van de Water J., Martínez-Cerdeño V. (2014). Embryonic Intraventricular Exposure to Autism-Specific Maternal Autoantibodies Produces Alterations in Autistic-like Stereotypical Behaviors in Offspring Mice. Behav. Brain Res..

[24] Sungur A.Ö., Vörckel K.J., Schwarting R.K.W., Wöhr M. (2014). Repetitive Behaviors in the Shank1 Knockout Mouse Model for Autism Spectrum Disorder: Developmental Aspects and Effects of Social Context. J. Neurosci. Methods.

[25] Cheaha D., Bumrungsri S., Chatpun S., Kumarnsit E. (2015). Characterization of in Utero Valproic Acid Mouse Model of Autism by Local Field Potential in the Hippocampus and the Olfactory Bulb. Neurosci. Res..

[26] Balaan C., Corley M.J., Eulalio T., Leite-ahyo K., Pang A.P.S., Fang R., Khadka V.S., Maunakea A.K., Ward M.A. (2019). Juvenile Shank3b Deficient Mice Present with Behavioral Phenotype Relevant to Autism Spectrum Disorder. Behav. Brain Res..

[27] Reith R.M., McKenna J., Wu H., Hashmi S.S., Cho S.H., Dash P.K., Gambello M.J. (2013). Loss of Tsc2 in Purkinje Cells Is Associated with Autistic-like Behavior in a Mouse Model of Tuberous Sclerosis Complex. Neurobiol. Dis..

[28] Schwartzer J.J., Careaga M., Onore C.E., Rushakoff J.A., Berman R.F., Ashwood P. (2013). Maternal Immune Activation and Strain Specific Interactions in the Development of Autism-like Behaviors in Mice. Transl. Psychiatry.

[29] Moy S.S., Riddick N.V., Nikolova V.D., Teng B.L., Agster K.L., Nonneman R.J., Young N.B., Baker L.K., Nadler J.J., Bodfish J.W. (2014). Repetitive Behavior Profile and Supersensitivity to Amphetamine in the C58/J Mouse Model of Autism. Behav. Brain Res..

[30] Shriberg L.D., Paul R., McSweeny J.L., Klin A., Cohen D.J., Volkmar F.R. (2001). Speech and Prosody Characteristics of Adolescents and Adults With High-Functioning Autism and Asperger Syndrome. J. Speech Lang. Hear. Res..

[31] Chabout J., Serreau P., Ey E., Bellier L., Aubin T., Bourgeron T., Granon S. (2012). Adult Male Mice Emit Context-Specific Ultrasonic Vocalizations That Are Modulated by Prior Isolation or Group Rearing Environment. PLoS ONE.

[32] Mun H.S., Lipina T.V., Roder J.C. (2015). Ultrasonic Vocalizations in Mice during Exploratory Behavior Are Context-Dependent. Front. Behav. Neurosci..

[33] Wöhr M., Roullet F.I., Hung A.Y., Sheng M., Crawley J.N. (2011). Communication Impairments in Mice Lacking Shank1: Reduced Levels of Ultrasonic Vocalizations and Scent Marking Behavior. PLoS ONE.

[34] Schmeisser M.J., Ey E., Wegener S., Bockmann J., Stempel A.V., Kuebler A., Janssen A.L., Udvardi P.T., Shiban E., Spilker C. (2012). Autistic-like Behaviours and Hyperactivity in Mice Lacking ProSAP1/Shank2. Nature.

[35] Yang M., Bozdagi O., Scattoni M.L., Wöhr M., Roullet F.I., Katz A.M., Abrams D.N., Kalikhman D., Simon H., Woldeyohannes L. (2012). Reduced Excitatory Neurotransmission and Mild Autism-Relevant Phenotypes in Adolescent Shank3 Null Mutant Mice. J. Neurosci..

[36] Ey E., Torquet N., Le Sourd A.M., Leblond C.S., Boeckers T.M., Faure P., Bourgeron T. (2013). The Autism ProSAP1/Shank2 Mouse Model Displays Quantitative and Structural Abnormalities in Ultrasonic Vocalisations. Behav. Brain Res..

[37] Greene-Colozzi E.A., Sadowski A.R., Chadwick E., Tsai P.T., Sahin M. (2014). Both Maternal and Pup Genotype Influence Ultrasonic Vocalizations and Early Developmental Milestones in Tsc2^+/−^ Mice. Epilepsy Res. Treat..

[38] Ju A., Hammerschmidt K., Tantra M., Krueger D., Brose N., Ehrenreich H. (2014). Juvenile Manifestation of Ultrasound Communication Deficits in the Neuroligin-4 Null Mutant Mouse Model of Autism. Behav. Brain Res..

[39] Bicks L.K., Koike H., Akbarian S., Morishita H. (2015). Prefrontal Cortex and Social Cognition in Mouse and Man. Front. Psychol..

[40] Ko J. (2017). Neuroanatomical Substrates of Rodent Social Behavior: The Medial Prefrontal Cortex and Its Projection Patterns. Front. Neural Circuits.

[41] Murugan M., Jang H.J., Park M., Miller E.M., Cox J., Taliaferro J.P., Parker N.F., Bhave V., Hur H., Liang Y. (2017). Combined Social and Spatial Coding in a Descending Projection from the Prefrontal Cortex. Cell.

[42] Banker S.M., Gu X., Schiller D., Foss-Feig J.H. (2021). Hippocampal Contributions to Social and Cognitive Deficits in Autism Spectrum Disorder. Trends Neurosci..

[43] Mehpara Farhat S., Mahboob A., Iqbal G., Ahmed T. (2017). Aluminum-Induced Cholinergic Deficits in Different Brain Parts and Its Implications on Sociability and Cognitive Functions in Mouse. Biol. Trace Elem. Res..

[44] Jupp B., Sawiak S.J., Van Der Veen B., Lemstra S., Toschi C., Barlow R.L., Pekcec A., Bretschneider T., Nicholson J.R., Robbins T.W. (2020). Diminished Myoinositol in Ventromedial Prefrontal Cortex Modulates the Endophenotype of Impulsivity. Cereb. Cortex.

[45] Sun L., Min L., Li M., Shao F. (2021). Juvenile Social Isolation Leads to Schizophrenia-like Behaviors via Excess Lactate Production by Astrocytes. Brain Res. Bull..

[46] Young D.M., Schenk A.K., Yang S.B., Jan Y.N., Jan L.Y. (2010). Altered Ultrasonic Vocalizations in a Tuberous Sclerosis Mouse Model of Autism. Proc. Natl. Acad. Sci. USA.

[47] Lidstone J., Uljarević M., Sullivan J., Rodgers J., McConachie H., Freeston M., Le Couteur A., Prior M., Leekam S. (2014). Relations among Restricted and Repetitive Behaviors, Anxiety and Sensory Features in Children with Autism Spectrum Disorders. Res. Autism Spectr. Disord..

[48] Baribeau D.A., Vigod S., Pullenayegum E., Kerns C.M., Mirenda P., Smith I.M., Vaillancourt T., Volden J., Waddell C., Zwaigenbaum L. (2020). Repetitive Behavior Severity as an Early Indicator of Risk for Elevated Anxiety Symptoms in Autism Spectrum Disorder. J. Am. Acad. Child Adolesc. Psychiatry.

[49] Ben-Itzchak E., Koller J., Zachor D.A. (2020). Characterization and Prediction of Anxiety in Adolescents with Autism Spectrum Disorder: A Longitudinal Study. J. Abnorm. Child Psychol..

[50] Rodgers J., Glod M., Connolly B., McConachie H. (2012). The Relationship between Anxiety and Repetitive Behaviours in Autism Spectrum Disorder. J. Autism Dev. Disord..

[51] Choi C.S., Gonzales E.L., Kim K.C., Yang S.M., Kim J.W., Mabunga D.F., Cheong J.H., Han S.H., Bahn G.H., Shin C.Y. (2016). The Transgenerational Inheritance of Autism-like Phenotypes in Mice Exposed to Valproic Acid during Pregnancy. Sci. Rep..

[52] Chang Y.C., Cole T.B., Costa L.G. (2018). Prenatal and Early-Life Diesel Exhaust Exposure Causes Autism-like Behavioral Changes in Mice. Part. Fibre Toxicol..

[53] Park S.M., Plachez C., Huang S. (2018). Sex-Dependent Motor Deficit and Increased Anxiety-like States in Mice Lacking Autism-Associated Gene Slit3. Front. Behav. Neurosci..

[54] Kouser M., Speed H.E., Dewey C.M., Reimers J.M., Widman A.J., Gupta N., Liu S., Jaramillo T.C., Bangash M., Xiao B. (2013). Loss of Predominant Shank3 Isoforms Results in Hippocampus-Dependent Impairments in Behavior and Synaptic Transmission. J. Neurosci..

[55] Zhou I.Y., Ding A.Y., Li Q., McAlonan G.M., Wu E.X. (2012). Magnetic Resonance Spectroscopy Reveals N-Acetylaspartate Reduction in Hippocampus and Cingulate Cortex after Fear Conditioning. Psychiatry Res. Neuroimaging.

[56] Gapp K., Corcoba A., van Steenwyk G., Mansuy I.M., Duarte J.M.N. (2017). Brain Metabolic Alterations in Mice Subjected to Postnatal Traumatic Stress and in Their Offspring. J. Cereb. Blood Flow Metab..

[57] Lefebvre E., Granon S., Chauveau F. (2020). Social Context Increases Ultrasonic Vocalizations during Restraint in Adult Mice. Anim. Cogn..

[58] Screven L.A., Dent M.L. (2019). Perception of Ultrasonic Vocalizations by Socially Housed and Isolated Mice. eNeuro.

[59] Werling D.M., Geschwind D.H. (2013). Sex Differences in Autism Spectrum Disorders. Curr. Opin. Neurol..

[60] Vignoli A., La Briola F., Peron A., Turner K., Vannicola C., Saccani M., Magnaghi E., Scornavacca G.F., Canevini M.P. (2015). Autism Spectrum Disorder in Tuberous Sclerosis Complex: Searching for Risk Markers. Orphanet J. Rare Dis..

[61] Hunt A., Shepherd C. (1993). A Prevalence Study of Autism in Tuberous Sclerosis. J. Autism Dev. Disord..

[62] Saré R.M., Lemons A., Figueroa C., Song A., Levine M., Smith C.B. (2020). Sex-Selective Effects on Behavior in a Mouse Model of Tuberous Sclerosis Complex. eNeuro.

[63] Zhao T., Huang G.B., Muna S.S., Bagalkot T.R., Jin H.M., Chae H.J., Chung Y.C. (2013). Effects of Chronic Social Defeat Stress on Behavior and Choline Acetyltransferase, 78-KDa Glucose-Regulated Protein, and CCAAT/Enhancer-Binding Protein (C/EBP) Homologous Protein in Adult Mice. Psychopharmacology.

[64] Gonçalves J., Violante I.R., Sereno J., Leitão R.A., Cai Y., Abrunhosa A., Silva A.P., Silva A.J., Castelo-Branco M. (2017). Testing the Excitation/Inhibition Imbalance Hypothesis in a Mouse Model of the Autism Spectrum Disorder: In Vivo Neurospectroscopy and Molecular Evidence for Regional Phenotypes. Mol. Autism.

[65] Zhao D., Xu X., Pan L., Zhu W., Fu X., Guo L., Lu Q., Wang J. (2017). Pharmacologic Activation of Cholinergic Alpha7 Nicotinic Receptors Mitigates Depressive-like Behavior in a Mouse Model of Chronic Stress. J. Neuroinflamm..

[66] Gitik M., Holliday E.D., Leung M., Yuan Q., Logue S.F., Tikkanen R., Goldman D., Gould T.J. (2018). Choline Ameliorates Adult Learning Deficits and Reverses Epigenetic Modification of Chromatin Remodeling Factors Related to Adolescent Nicotine Exposure. Neurobiol. Learn. Mem..

[67] Kim S., Hong K.B., Kim S., Suh H.J., Jo K. (2020). Creatine and Taurine Mixtures Alleviate Depressive-like Behaviour in Drosophila Melanogaster and Mice via Regulating Akt and ERK/BDNF Pathways. Sci. Rep..

[68] Claghorn G.C., Thompson Z., Wi K., Van L., Garland T. (2017). Caffeine Stimulates Voluntary Wheel Running in Mice without Increasing Aerobic Capacity. Physiol. Behav..

[69] Corcoba A., Gruetter R., Do K.Q., Duarte J.M.N. (2017). Social Isolation Stress and Chronic Glutathione Deficiency Have a Common Effect on the Glutamine-to-Glutamate Ratio and Myo-Inositol Concentration in the Mouse Frontal Cortex. J. Neurochem..

[70] Bottomley P. (1987). Spatial Localization in NMR Spectroscopy in vivo. Ann. N. Y. Acad. Sci..

[71] Tkáč I., Starčuk Z., Choi I.-Y., Gruetter R. (1999). In Vivo 1 H NMR Spectroscopy of Rat Brain at 1 Ms Echo Time. Magn. Reson. Med..

[72] Provencher S.W. (2001). Automatic Quantitation of Localized in Vivo 1H Spectra with LCModel. NMR Biomed..

[73] Cox K.H., Rissman E.F. (2011). Sex Differences in Juvenile Mouse Social Behavior Are Influenced by Sex Chromosomes and Social Context. Genes Brain Behav..

[74] Terranova M.L., Laviola G. (2005). Scoring of Social Interactions and Play in Mice During Adolescence. Curr. Protoc. Toxicol..

[75] Stanford School of Medicine Mouse Species Ethogram. http://mousebehavior.org/ethogram/.

[76] Angoa-Pérez M., Kane M.J., Briggs D.I., Francescutti D.M., Kuhn D.M. (2013). Marble Burying and Nestlet Shredding as Tests of Repetitive, Compulsive-like Behaviors in Mice. JoVE J. Vis. Exp..

