# Supplementary material for: Sex-Dependent Social and Repetitive Behavior and Neurochemical Profile in Mouse Model of Autism Spectrum Disorder
Source: Metabolites. 2022 Jan 12;12(1):71. doi: 10.3390/metabo12010071 (PMC8778172; doi:10.3390/metabo12010071)
Supplement: Supplementary file 1 [file metabolites-12-00071-s001.zip › metabolites-1537306-supplementary.pdf]

metabolites

Article

Sex-dependent social and repetitive behavior and neurochemical profile in mouse model of autism spectrum disorder

Helena Ferreira [1,2], Ana Catarina Sousa [3], José Sereno [2,4], João Martins [2,4], Miguel Castelo-Branco [1,2,4*] and Joana Gonçalves [2,4*]

1 University of Coimbra, Faculty of Medicine, Portugal
2 University of Coimbra, Coimbra Institute for Biomedical Imaging and Translational Research (CIBIT), Portugal
3 University of Coimbra, Faculty of Sciences and Technology, Portugal
4 University of Coimbra, Institute of Nuclear Sciences Applied to Health (ICNAS), Portugal
* Correspondence: MCB: mcbranco@fmed.uc.pt; JG: jgoncalves@fmed.uc.pt; Tel.: +351 239 488510

Supplementary Table S1. ^{1}H-magnetic resonance spectroscopy (MRS) analysis of cortical neurochemical profile.

Brain metabolite	Prefrontal cortex			
	Male		Female	
	WT	*Tsc2*$^{+/-}$	WT	*Tsc2*$^{+/-}$
GABA	2.64 ± 0.1132	2.60 ± 0.1167	3.38 ± 0.2118	**2.57 ± 0.1685** $^{\#}$
Glutamate	12.14 ± 0.3246	12.54 ± 0.5710	10.66 ± 0.7597	**13.43 ± 0.5382** $^{\#}$
Glutamine	4.57 ± 0.2307	5.09 ± 0.4838	4.27 ± 0.3094	4,20 ± 0.2505
Alanine	4.52 ± 0.0606	**2.43 ± 0.1913** *	5.31 ± 1.4300	**5.69 ± 0.6611** $^{++}$
Total choline	1.56 ± 0.0416	**1.77 ± 0.0412** *	1.50 ± 0.0801	**1.80 ± 0.0206** $^{\#\#}$
NAA+NAAG	10.42 ± 0.1361	10.29 ± 0.3037	10.24 ± 0.2376	9.91 ± 0.3070
Total creatine	8.97 ± 0.1569	9.06 ± 0.1186	8.62 ± 0.2667	9.21 ± 0.1817
Glutathione	2.50 ± 0.1113	**2.11 ± 0.0606** *	2.76 ± 0.1405	2.39 ± 0.1185
Inositol	4.71 ± 0.2387	4.99 ± 0.1379	5.04 ± 0.4906	**6.17 ± 0.2288** $^{+}$
Lactate	3.48 ± 0.7489	3.22 ± 0.2109	7.11 ± 1.0330	6.08 ± 1.0610
Taurine	12.26 ± 0.3411	11.65 ± 0.0996	11.75 ± 0.7583	**13.07 ± 0.4239** $^{+}$

* Male WT vs Male *Tsc2*$^{+/-}$; $^{\#}$ Female WT vs Female *Tsc2*$^{+/-}$; $^{+}$ Male *Tsc2*$^{+/-}$ vs Female *Tsc2*$^{+/-}$

Supplementary Table S2. ^{1}H-magnetic resonance spectroscopy (MRS) analysis of hippocampal neurochemical profile.

Brain metabolite	Hippocampus			
	Male		Female	
	WT	*Tsc2*$^{+/-}$	WT	*Tsc2*$^{+/-}$
GABA	2.66 ± 0.1303	2.75 ± 0.1102	2.88 ± 0.2150	2.78 ± 0.1297
Glutamate	9.91 ± 0.3096	10.10 ± 0.2078	10.14 ± 0.4660	9.88 ± 0.3423
Glutamine	6.05 ± 0.6076	5.18 ± 0.2539	5.21 ± 0.2824	5.60 ± 0.1189
Alanine	2.75 ± 0.0835	2.90 ± 0.3880	2.35 ± 0.1917	2.48 ± 0.1539
Total choline	1.65 ± 0.0099	**1.87 ± 0.0698** *	**1.93 ± 0.0699** $^{\$\$}$	1.82 ± 0.0361
NAA+NAAG	8.85 ± 0.2010	9.48 ± 0.1503	8.918 ± 0.2360	**8.603 ± 0.2047** $^{+}$
Total creatine	9.423 ± 0.2310	9.785 ± 0.1470	9.259 ± 0.1112	9.296 ± 0.0650
Glutathione	2.25 ± 0.1109	2.41 ± 0.1767	2.20 ± 0.1426	2.00 ± 0.0647
Inositol	4.77 ± 0.1217	**5.67 ± 0.1246** **	5.42 ± 0.2458	5.47 ± 0.1433
Lactate	3.39 ± 0.1770	**4.51 ± 0.2463** *	3.50 ± 0.3303	3.57 ± 0.1376
Taurine	10.81 ± 0.2973	**11.79 ± 0.1872** *	10.98 ± 0.2062	**10.80 ± 0.1976** $^{+}$

* Male WT vs Male *Tsc2*$^{+/-}$; $^{\#}$ Female WT vs Female *Tsc2*$^{+/-}$; $^{\$}$ Male WT vs Female WT; $^{+}$ Male *Tsc2*$^{+/-}$ vs Female *Tsc2*$^{+/-}$

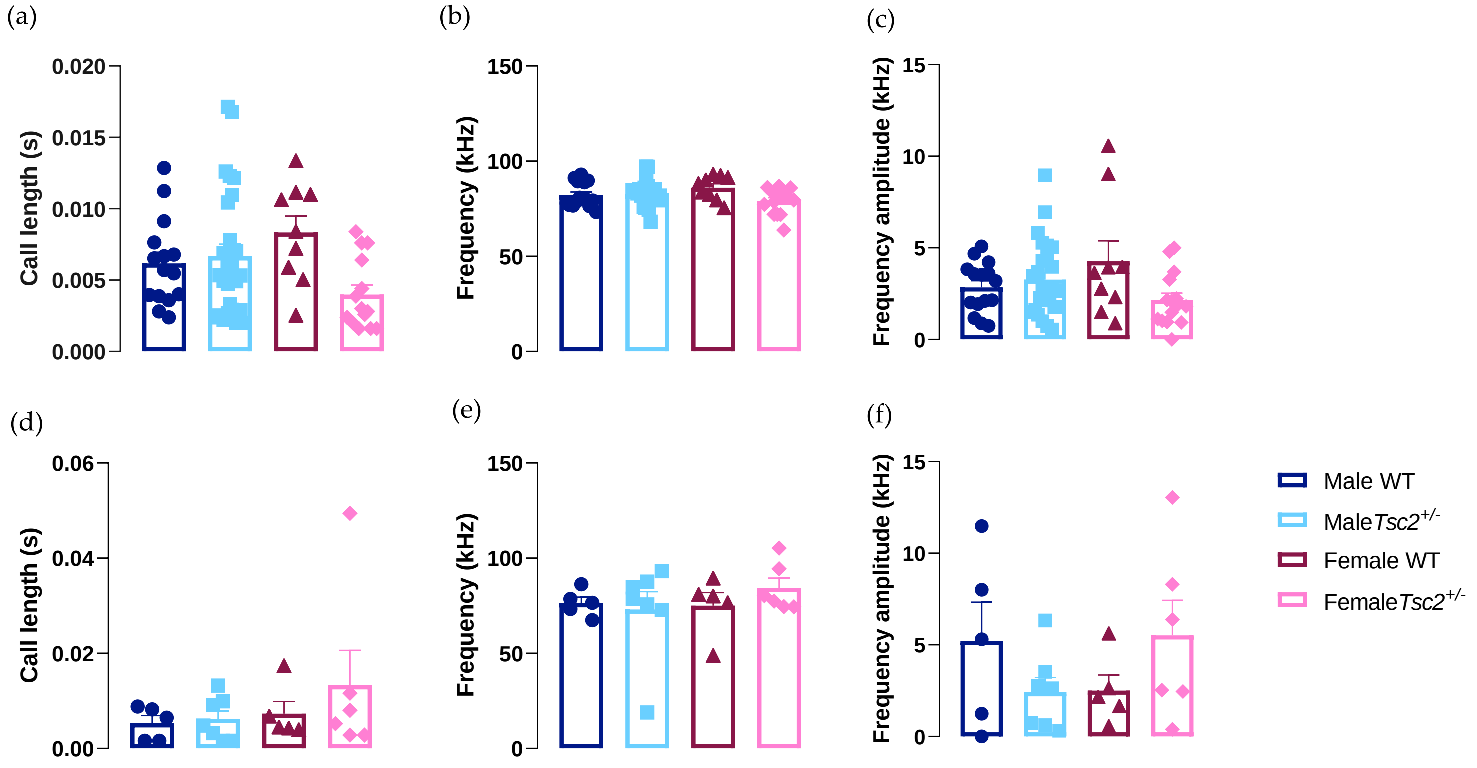

Supplementary Figure S1. (**a**) Length, (**b**) principal frequency and (**c**) frequency amplitude of USVs produced during social behavior task, and (**d**) length, (e) principal frequency and (**f**) frequency amplitude of USVs produced during RRB-eliciting task.

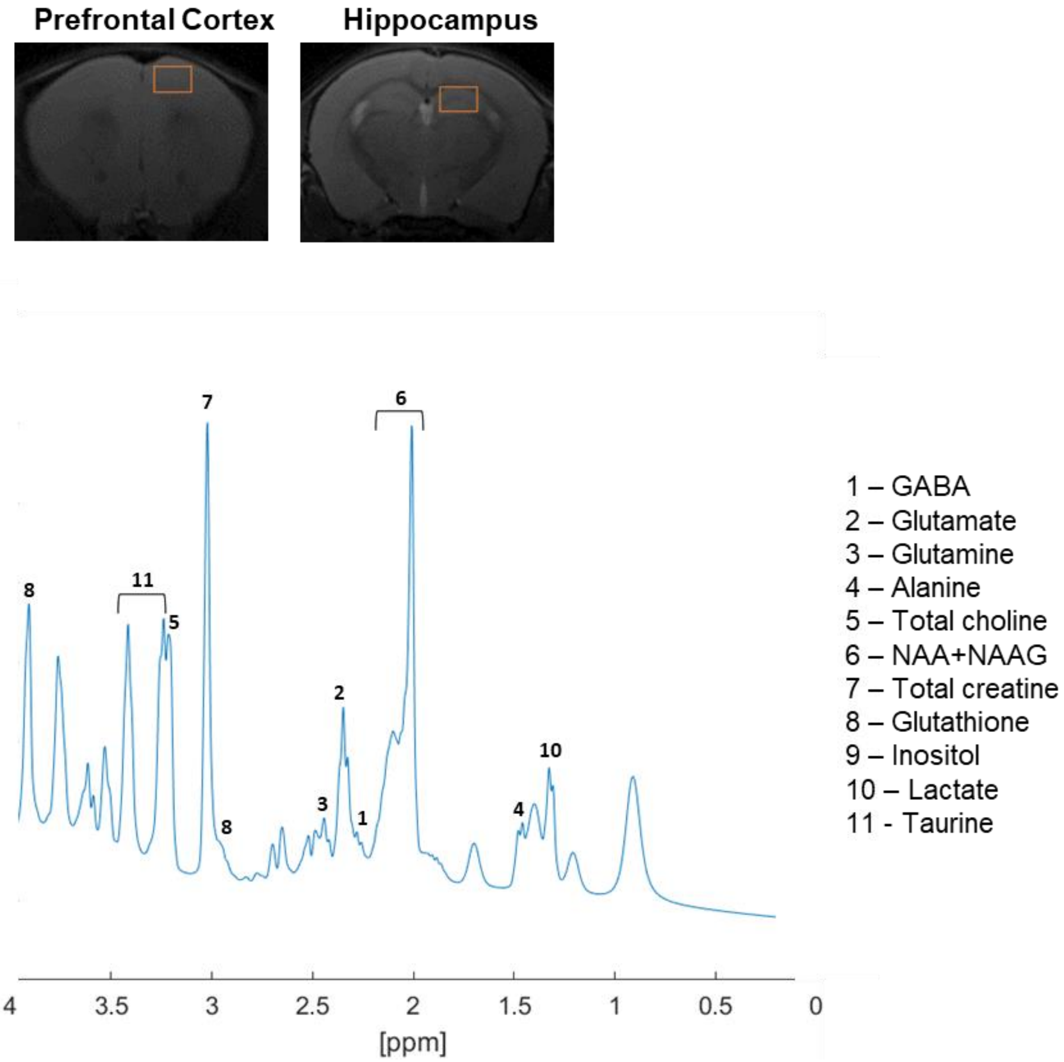

Supplementary Figure S2. Localized ^{1}H NMR voxel (orange square) in the (**a**) prefrontal cortex and (**b**) hippocampus of a 60-days old mouse acquired with 9.4T MR pre-clinical scanner (Bruker Biospec, Billerica MA). Representative fast-spin-echo image of a spectrum was used to show typical location of the volume of interest for spectroscopy.
